# Leaf area index estimation model for UAV image hyperspectral data based on wavelength variable selection and machine learning methods

**DOI:** 10.1186/s13007-021-00750-5

**Published:** 2021-05-03

**Authors:** Juanjuan Zhang, Tao Cheng, Wei Guo, Xin Xu, Hongbo Qiao, Yimin Xie, Xinming Ma

**Affiliations:** 1 Science College of Information and Management, Henan Agricultural University, #63 Nongye Road, Zhengzhou, 450002 Henan China; 2 Collaborative Innovation Center of Henan Grain Crops, Henan Agricultural University, #63 Nongye Road, Zhengzhou, 450002 Henan China; 3 College of agronomy, Henan Agricultural University, #63 Nongye Road, ZhengZhou, Henan 450002 China

**Keywords:** Winter wheat, Leaf area index, Unmanned aerial vehicle, Hyperspectral imaging data, Characteristic bands, Machine learning, Model

## Abstract

**Background:**

To accurately estimate winter wheat leaf area index (LAI) using unmanned aerial vehicle (UAV) hyperspectral imagery is crucial for crop growth monitoring, fertilization management, and development of precision agriculture.

**Methods:**

The UAV hyperspectral imaging data, Analytical Spectral Devices (ASD) data, and LAI were simultaneously obtained at main growth stages (jointing stage, booting stage, and filling stage) of various winter wheat varieties under various nitrogen fertilizer treatments. The characteristic bands related to LAI were extracted from UAV hyperspectral data with different algorithms including first derivative (FD), successive projections algorithm (SPA), competitive adaptive reweighed sampling (CARS), and competitive adaptive reweighed sampling combined with successive projections algorithm (CARS_SPA). Furthermore, three modeling machine learning methods including partial least squares regression (PLSR), support vector machine regression (SVR), and extreme gradient boosting (Xgboost) were used to build LAI estimation models.

**Results:**

The results show that the correlation coefficient between UAV and ASD hyperspectral data is greater than 0.99, indicating the UAV data can be used for estimation of wheat growth information. The LAI bands selected by using different algorithms were slightly different among the 15 models built in this study. The Xgboost model using nine consecutive characteristic bands selected by CARS_SPA algorithm as input was proved to have the best performance. This model yielded identical results of coefficient of determination (0.89) for both calibration set and validation set, indicating a high accuracy of this model.

**Conclusions:**

The Xgboost modeling method in combine with CARS_SPA algorithm can reduce input variables and improve the efficiency of model operation. The results provide reference and technical support for nondestructive and rapid estimation of winter wheat LAI by using UAV.

## Background

Leaf area index (LAI), which is defined as half of the all-sided leaf area per unit ground area [1], is a key biophysical parameter to determine the photosynthesis, respiration, and transpiration of vegetation canopy [2, 3]. Winter wheat (*Triticum aestivum* L.) is a main food crop in China. It is of great importance to obtain the winter wheat LAI rapidly and effectively to monitor wheat growth, manage water and fertilizer application, and predict the yield. Destructive methods for measuring winter wheat LAI normally provide more precise results, but the assessment is time-consuming, labor-intensive, and expensive. The basic requirement of modern agriculture is to conduct real-time, fast, and accurate measurement of winter wheat LAI in the field, yet it is difficult to actualize. Remote sensing is a reliable, fast, and non-destructive way to monitor growth parameters of crops. Specifically, the newly emerged low-altitude remote sensing detection technology based on UAV exhibits characteristics of high spatial resolution, strong timeliness, low cost, and low flight altitude that does not require a flight permit. This technology can fill the gap between ground-based monitoring and satellite remote sensing of dynamically monitoring crop growth at multiple scales, therefore, has been widely used in precision agriculture [4–9].

UAV remote sensing platform can carry digital cameras featured with simultaneous interpreting and multispectral or hyperspectral sensors. The accuracy of crop phenotypic information by remote sensing are varied due to inconsistent performance among devices [10]. Modern hyperspectral sensors that continuously cover all spectral regions can successfully reflect the characteristics of crops. UAV has been used in earlier studies to analyze the characteristic bands related to LAI. The estimation models of wheat LAI built based on vegetation indices (VIs) are used to extract wheat growth information from low- altitude UAV hyperspectral data. For example, by combining UAV-based hyperspectral data, Fu et al. [11] used Red Edge Soil Adjusted Vegetation Index (RESAVI) to effectively invert winter wheat LAI. Xie et al. [12] estimated the wheat LAI using six spectral indices of UAV hyperspectral data. However, spectral indices show different degrees of saturation [13, 14], and their universality and accuracy are easily disturbed by external factors [15].

In recent years, some deep learning algorithms such as convolutional neural networks (CNN) and machine learning algorithms such as support vector machine regression (SVR), partial least squares regression (PLSR), neural network, and random forest (RF) have been applied to agricultural condition monitoring, plant disease and insect monitoring, wheat ear identification and other aspects, and have shown good results. For example, Li et al. [16, 17] used CNN to carry out identification and monitoring of plant diseases and insect pests. Xu et al. [18] used CNN to achieve accurate segmentation and recognition of the number of wheat ears. In the area of UAV spectral monitoring, by using UAV hyperspectral data, Gao et al. [19] extracted spectral features based on UAV hyperspectral data and constructed the winter wheat LAI estimation model using PLSR. Yue et al. [20] constructed estimation model of wheat LAI by using VIs, RF and PLSR. In aforementioned studies, the extracted spectral parameters and ML algorithms are combined to construct inversion models of physiological parameters. In recent years, algorithms for spectral features extraction such as principal component analysis (PCA), variable projection importance (VIP), genetic algorithm (GA), and continuous projection algorithm (SPA) have been widely used in studies of ground monitoring or satellite remote sensing [21, 22]. These algorithms can effectively remove the redundancy in hyperspectral data, thus reduce the risk of overfitting, and finally obtained a model of robust and high prediction accuracy [23–25]. Due to the inconsistency in performance of sensors, spectral data obtained are varied. However, at present, limited research has been conducted on dimensionality reduction and characteristic band extraction based on UAV hyperspectral imaging data and ML methods for winter wheat LAI estimation.

In this study, the UAV hyperspectral data, ASD non-imaging hyperspectral data, and LAI measurements of winter wheat at various key growth stages were obtained. Various variables extraction algorithms were used to extract characteristic bands related to LAI. Next, three ML methods (PLSR, SVR, and Xgboost) were employed to construct estimation models of winter wheat LAI based on selected spectral variables. Last, these models were comprehensively compared and a most suitable model for estimating winter wheat LAI was determined. This study provides the methodology and technical support for UAV remote sensing on winter wheat LAI estimation.

## Materials and methods

### Study area and experimental design

**Fig. 1:**
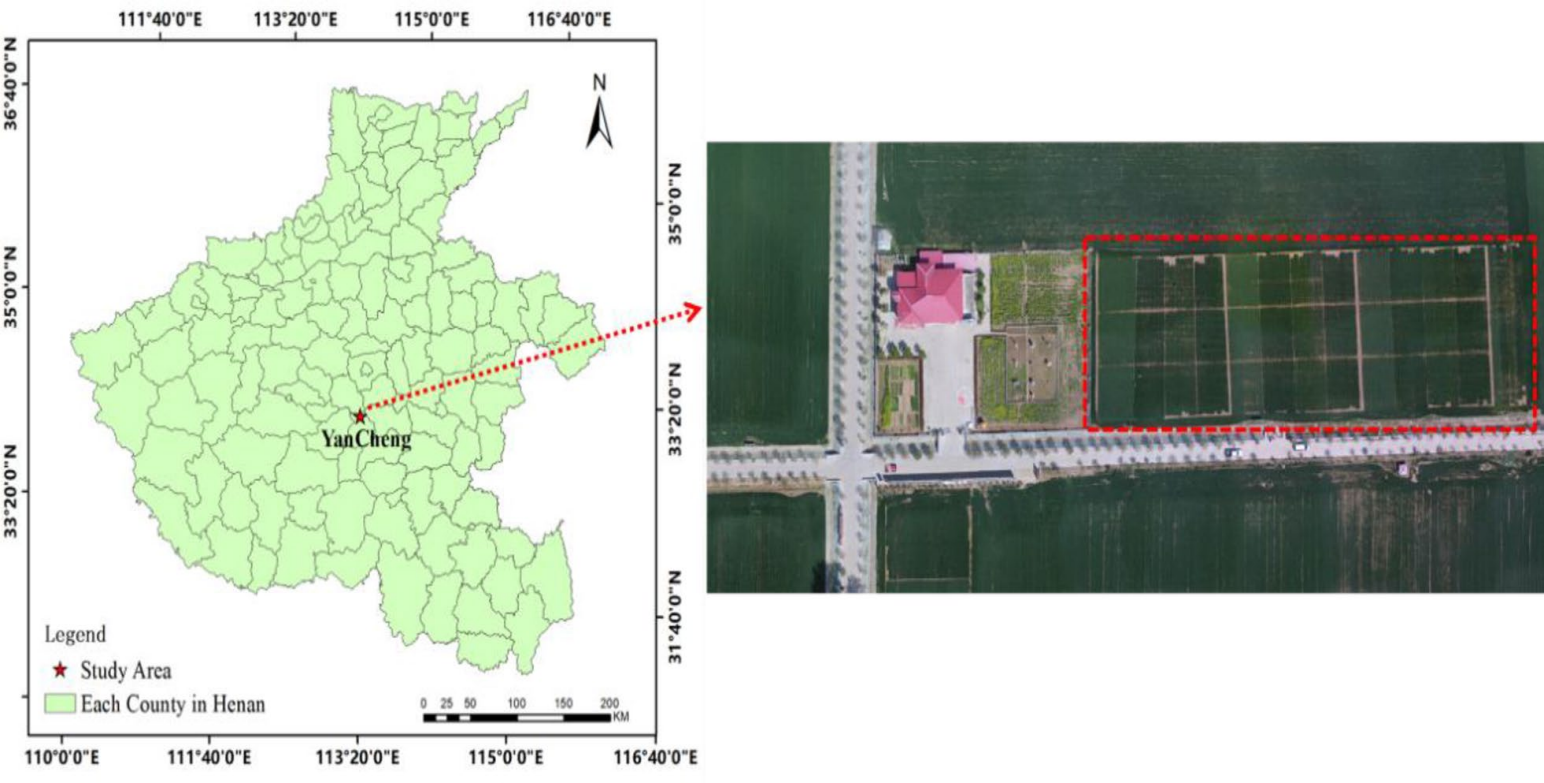
Study area location

**Fig. 2:**
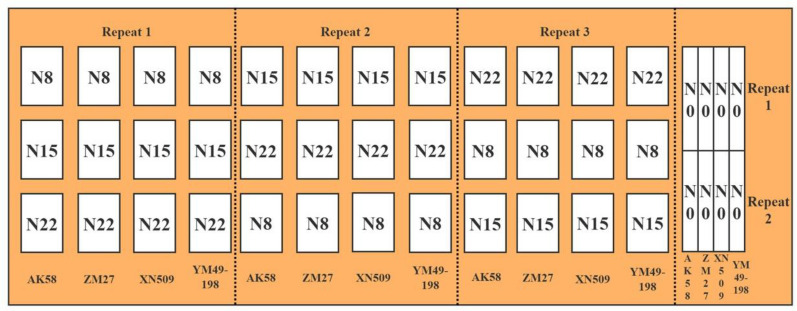
Experimental design. AK58, ZM27, XN509, and YM49-198 represent different winter wheat cultivars. AK58: Aikang 58; ZM27: Zhoumai 27; XN509: Xinong509; YM49-198: Yumai49-198. N0, N8, N15, and N22 represent nitrogen treatments of 0, 120, 225, and 330 kg hm^−2^, respectively

Experiments were conducted from 2017 to 2018 at Xindian Regional Test Station, Yancheng District, Luohe City, Henan Province, China (113°53′1″E, 33°41′60″N) (Fig. 1). The area has a warm wet monsoon climate, with precipitation mostly occurring in summer and autumn, an average annual temperature of about 14.6 °C. The contents of soil organic matter, total nitrogen, alkali hydrolyzed nitrogen, available phosphorus, and available potassium are 13.33 g kg^−1^, 1.02 g kg^−1^, 92.33 g kg^−1^, 54.02 mg kg^−1^, and 299 mg kg^−1^, respectively. The winter wheat varieties used in this study include Zhoumai 27 (ZM27), Yumai 49–198 (YM49-198), Xinong 509 (XN509), and Aikang 58 (AK58). Winter wheats were planted in 44 plots (each of size 16 m × 9 m) including three repeats (Fig. 2). Different nitrogen was used in each plot. A set of four nitrogen treatments were used: 0 (N0), 120 kg.hm^−2^ (N8), 225 kg.hm^−2^ (N15), and 330 kg.hm^−2^ (N22). The ratio of base fertilizer topdressing is 6:4. The base fertilizer was applied before sowing and the topdressing was applied at the jointing stage. Winter wheats were machine-sown on October 23, 2017, at a sowing rate of 180 kg hm^−2^. Other cultivation and management measures were generally the same as those applied in high yield wheat fields. The specific design is shown in Fig. 2.

### Data collection and index determination

All experimental data including UAV-based hyperspectral imaging data, field hyperspectral reflectance, and wheat LAI, were collected at three key growth stages of winter wheat in 2018. The specific dates for data collection were March 11 (jointing stage), April 8 (booting stage), and May 12 (filling stage).

#### UAV-based imaging hyperspectral data

**Fig. 3:**
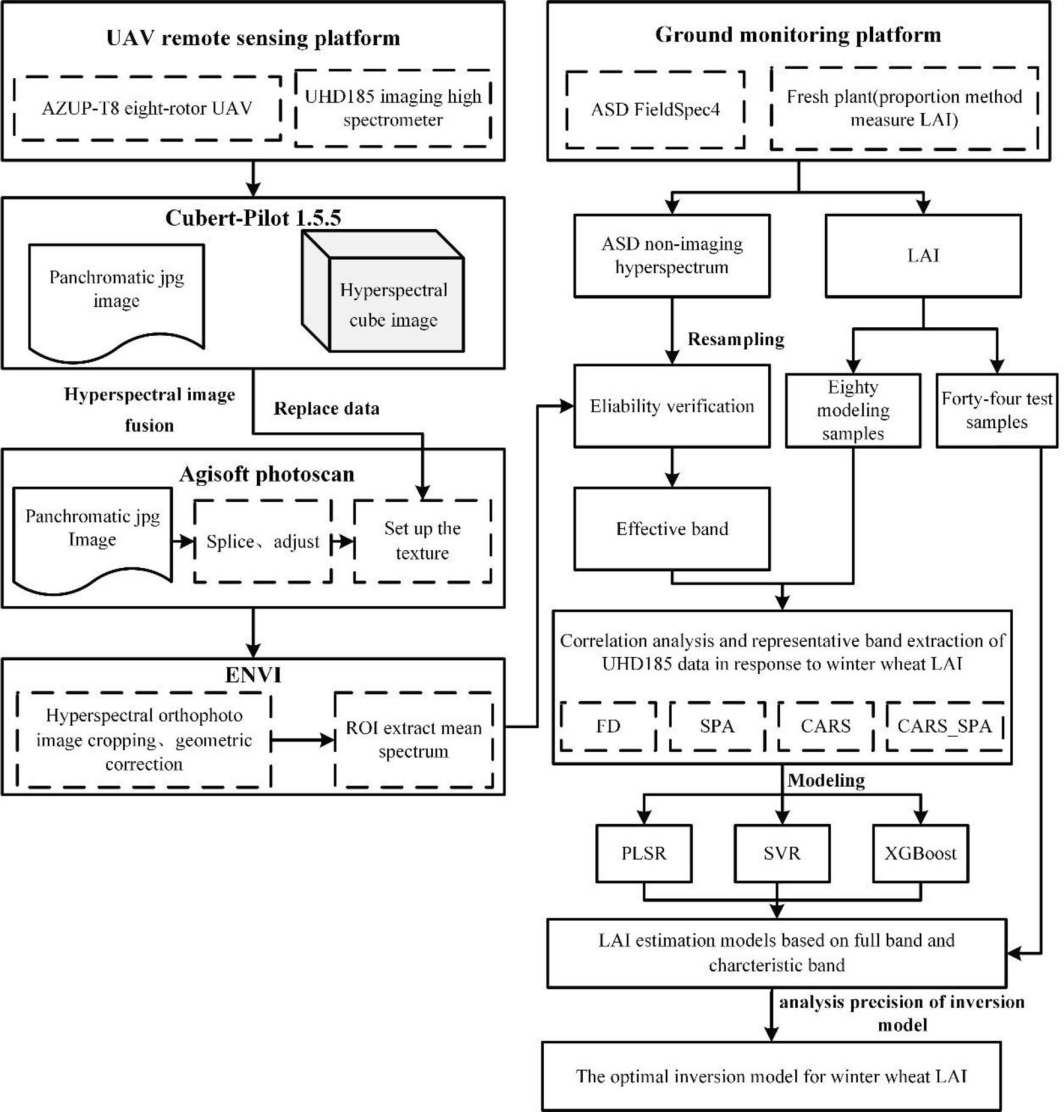
Flow chart of winter wheat LAI estimation modelling method

The UAV remote sensing platform is composed of an AZUP-T8 eight-propeller UAV (XIRO company, China), and a UHD185 high-resolution imaging spectrometer (Cubert, Germany). The UHD185 acquires wavelengths from the visible to the near-infrared (450–950 nm) from 125 spectral bands. The sampling interval of the hyperspectral data is 4 nm, and the spectral resolution is 8 nm. The data acquired by the UHD185 airborne hyperspectrometer contained hyperspectral cube images with a spatial resolution of 21 cm and panchromatic images (in.jpg format) with a spatial resolution of 1 cm. Hyperspectral data acquisition was carried out on cloudless and windless days. The reference plate was used to calibrate UHD185 before data collection. The height, moving speed, course overlap, and side overlap were set as 50 m, 6 m s^−1^, 80%, and 60%, respectively. The data processing flow is shown in Fig. 3.

#### Measurement of field hyperspectral reflectance

Canopy reflectance measurement of each plot was collected with an ASD FieldSpec4 Portable high-resolution spectrometer (ASD Inc., USA) with the spectral range of 350–2500 nm. ASD measurement was carried out before UAV data acquisition. The measurements were taken 1 m above the canopy with a 25° field of view optic. Three spectral measurements were acquired per plot (10 spectra as a sampling interval) and averaged to a single measurement for further analysis. The sampling interval of the hyperspectral data is 1.4 nm at 350–1000 nm, and 2 nm at 1000–2500 nm, and the spectral resolutions are 3 and 2 nm, respectively. Standard whiteboard correction was performed before and after the measurement. The exact position of each plot was located by a GPS device.

#### Measurement of LAI

Winter wheat LAIs were measured at fixed sample points of each plot. Ten winter wheat plants were randomly sampled from each plot, immediately sealed into a paper bag, and brought back to the laboratory for separation of stems and leaves. LAIs were measured according to the method proposed by Feng et al. [26].

### Data analysis

A total of 132 winter wheat LAIs were collected in this study. Eight were removed due to test errors, and the resulting 124 LAIs were used for the following data analysis. To evaluate the models’ robustness of temporal variation, the LAIs of area 1 and 3 (80 samples) were used as the calibration set, and the LAIs of area 2 (44 samples) were used as the standalone validated set. The LAI data are summarized in Table 1.

**Table 1:** Summary of leaf area index (LAI) of winter wheat

Sample type	Sample number	Maximum value	Minimum value	Mean value	Standard deviation	Coefficient of variation
Total sample	124	8.38	1.78	5.43	1.70	0.32
Calibration set	80	8.38	1.78	5.33	1.74	0.33
Validation set	44	8.24	3.10	5.62	1.62	0.30

The characteristic bands of LAI in UAV hyperspectral data were extracted with various algorithms including the first derivative (FD), successive projections algorithm (SPA), competitive adaptive reweighed sampling (CARS) and competitive adaptive reweighed sampling combined with successive projections algorithm (CARS_SPA). In combine with the full spectrum information, three ML methods including partial least squares regression (PLSR), support vector machine regression (SVR), and extreme gradient boosting (Xgboost) were used to construct LAI estimation models. The flowchart of UAV hyperspectral imaging data processing and data analysis for constructing winter wheat LAI estimation model is presented in Fig. 3.

#### Algorithms for spectral variables extraction

Four algorithms for spectral variables extraction were used in the present study. (1) FD can effectively remove the interference of linear and near-linear background noise in the raw spectrum, enhancing spectral characteristic differences. (2) SPA selects the combination of variables with minimum redundancy from the spectral matrix to minimize the collinearity among variables, resulting to a great reduction in the number of variables used as input for modeling. Therefore, the complexity of the model is reduced whereas the stability and accuracy of the model are improved [27]. (3) CARS is a new variable selection algorithm, which follows the principle of “survival of the fittest”. The exponential decreasing function (EDF) and adaptive reweighting sampling (ARS) are used to remove the wavelength variables with low absolute value of regression coefficients in PLSR model. After that, root mean square error of cross-validation (RMSECV) calculation are used to select important variables with good stability in several iterations, and a final subset of variables is obtained. The subset with the lowest RMSEV is considered as the optimal variable subset [28]. (4) CARS_SPA. By combining the advantages of the two algorithms (SPA and CARS) [29], the CARS_SPA algorithm not only minimize the spectral variable redundancy, but also reduces the interference of invalid variables during the SPA calculation process.

#### Modeling methods

Three ML methods were used for data mining and matrix recognition. (1) PLSR is a regression modeling method to predict a set of dependent variables from a set of independent variables. It combines features of principal component analysis, canonical correlation analysis, and multiple regression analysis. PLSR can solve the multi-collinearity among independent variables and is suitable for small sample datasets. (2) SVR. The use of Lagrangian multipliers for data regression analysis is an extension of support vector machine classification to solve regression problems. It has a strong learning ability from small datasets and solves high-dimensional and nonlinear problems by transforming them into linear problems through nonlinear transformation. In this study, the Gaussian kernel function is used as the kernel function, and the GridSerachCV function is used to discover the optimal parameters (penalty coefficient cost and gamma). (3) Xgboost is a new efficient ensemble learning algorithm proposed by Chen [30]. It is an improved algorithm of gradient boosting, and uses the Taylor expansion to obtain the second derivative as independent variable. By separating the selection of loss function from the optimization of model algorithm and parameter selection, the applicability of Xgboost is increased, which makes it select loss function on demand. A strategy similar to RF is adopted to support data sampling, and make full use of the advantages of multi-core CPU parallel computing, which greatly improves the operation speed and prediction accuracy of the model [31]. The GridSerachCV function is used to discover the optimal kernel parameters, the main kernel parameters are as follows: n_estimators, the maximum depth of the tree (max_depth), regularization parameters (min_child_weight), gamma, sampling method (subsample and colsample_bytree), and learning_rate.

### Accuracy evaluation

The accuracy of the LAI estimation models was evaluated using the coefficient of determination (*R*^*2*^), root mean square error (RMSE), and relative percent deviation (RPD). The formulas are as follows:

1 $${R}^{2}=\frac{{\sum }_{i=1}^{n}(xi-\overline{x}{)}^{2}\times (yi-\overline{y}{)}^{2}}{{\sum }_{i=1}^{n}(xi-\overline{x}{)}^{2}\times {\sum }_{i=1}^{n}(yi-\overline{y}{)}^{2}}$$

2 $$RMSE=\sqrt{\frac{{\sum }_{i=1}^{n}(yi-xi{)}^{2}}{n}}$$

3 $$RPD=\sqrt{\frac{{\sum }_{i=1}^{n}(xi-{\overline{x})}^{2}}{n-1}}/RMSE$$

where, $xi$, $\overline{x}$, $yi$, and $\overline{y}$ are the measured LAI, the mean measured LAI, the LAI predicted by the model, and the mean LAI predicted by the model, respectively; n is the number of data points. Larger *R*^*2*^ values indicate a better model fit, while a smaller RMSE indicates a higher model accuracy. RPD can reflect the prediction performance of the model. Briefly, when 1.0 < RPD < 1.4, the prediction performance of the model is poor; when 1.4 < RPD < 1.8, the model can be used for correlation assessment; when 1.8 < RPD < 2.0, the model can be used for quantitative prediction; when 2.0 < RPD < 2.5, a more accurate quantitative prediction can be achieved; when RPD > 2.5, the prediction performance of the model is better.

## Results and analysis

### Reliability verification of UAV hyperspectral imaging data

**Fig. 4:**
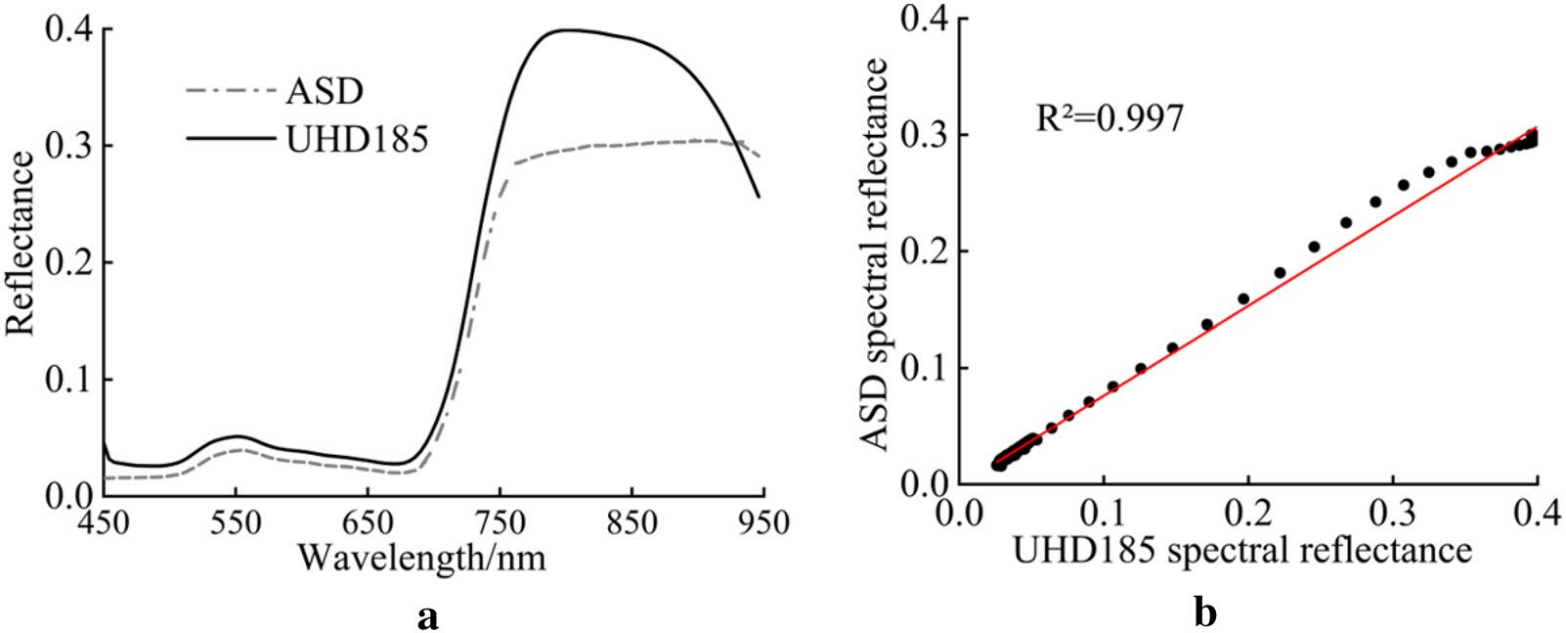
Reliability verification of UHD185 hyperspectral data. **a** Comparison of spectral reflectance curves of UHD185 and resampled ASD, **b** Correlation between UHD185 and resampled ASD spectral reflectance

In order to verify the reliability of UAV hyperspectral imaging data, the winter wheat hyperspectral data collected by ASD were resampled into UHD185 bands to calculate the average reflectance for each plot. Correlation analysis showed that the spectral reflectance of UHD185 and ASD are in high consistence at spectral bands between 458 and 830 nm, overlapping in both green peak position and red edge region (Fig. 4a). At spectral bands between 830 and 950 nm, the spectral reflectance of UHD185 gradually decreased, while that of ASD deviated from UHD185 and exhibited a flat curve (Fig. 4a). This may be due to relatively high noise between 830 and 950 nm since this range is close to the edge of UHD185 sensor spectrum. We compared spectral reflectance of UHD185 and ASD at 458–830 nm and found a high correlation (*R*^*2*^ > 0.99) between them (Fig. 4b). These results indicate that the spectral reflectance of UHD185 is reliable between 458 and 830 nm (3–96 wavebands) and can be used to estimate winter wheat LAI.

### Changes in LAI and spectrum under different nitrogen treatments at different growth stages

**Fig.5:**
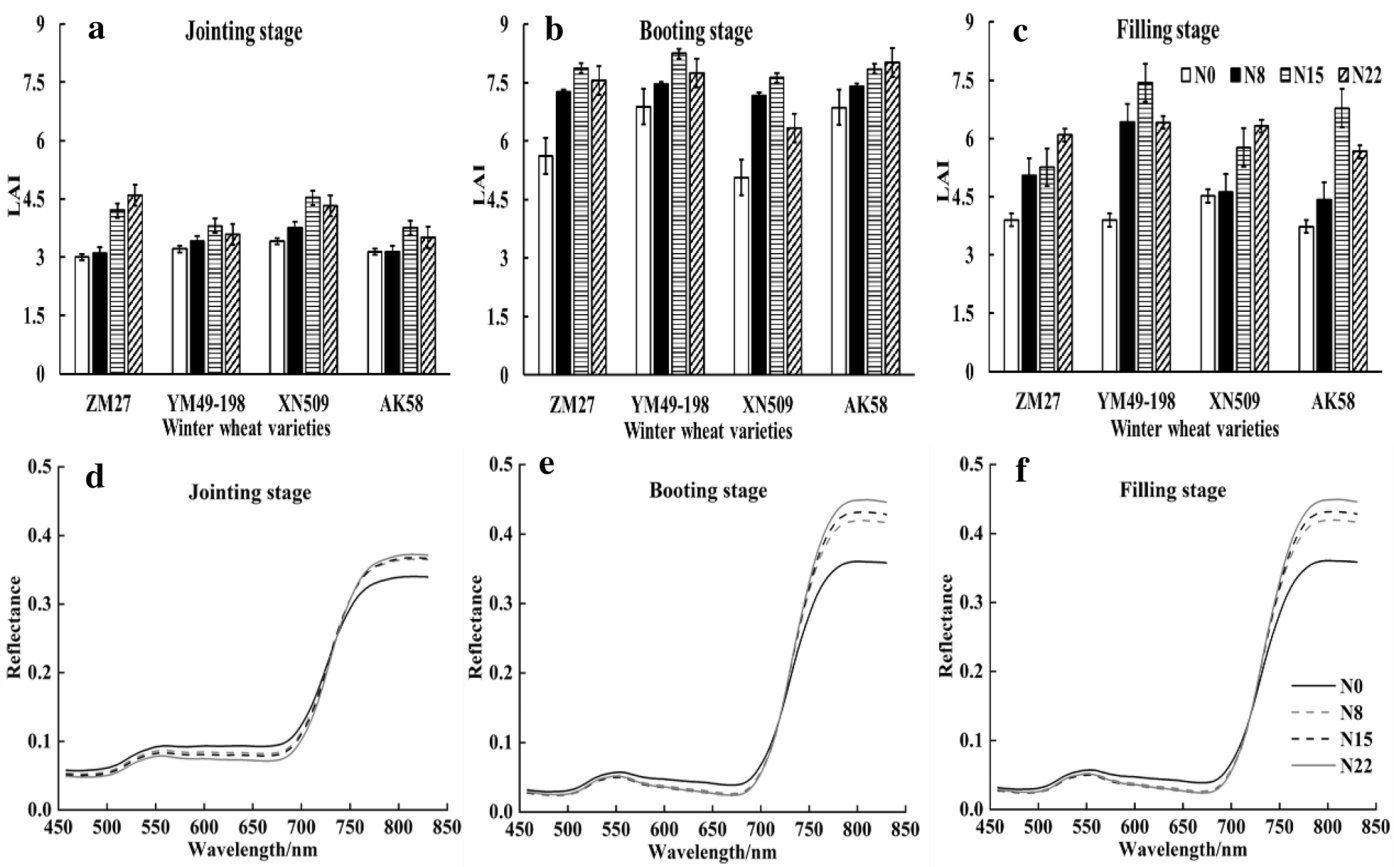
Changes in LAI and spectrum of winter wheat varieties under various nitrogen treatments at distinct growth stages

The four winter wheat varieties responded differently to various N treatments at distinct growth stages (Fig. 5a–c). Under various N treatments, the maximum LAI values were observed under N15 and N22. With the wheat growth developed, LAI first increased and then decreased, with the maximum LAI value obtained at the booting stage. The changes in canopy spectral features at 458–830 nm under various N treatments were further analyzed, and the overall trends of canopy spectra were consistent among wheat varieties. The changes in canopy characteristics of YM49-198 are shown in Fig. 5d–f. The overall trend of canopy spectral reflectance was similar under various N treatments: the spectral reflectance was low at 458–730 nm (visible region) and was high in the near-infrared region; an absorption valley (red valley) appeared near 674 nm, and the spectral reflectance rose sharply at 690–790 nm (red-edge region). At 458–730 nm (visible region), the spectral reflectance decreased with increasing N concentrations, showing N0 > N8 > N15 > N22. The difference in spectral reflectance among various N treatments was more significant in the near-infrared region than in the visible region, and the spectral reflectance increased with increasing N concentrations, showing N22 > N15 > N8 > N0. The overall trend was consistent among different growth stages.

### Correlation between winter wheat LAI and UAV hyperspectral imaging data

**Fig. 6:**
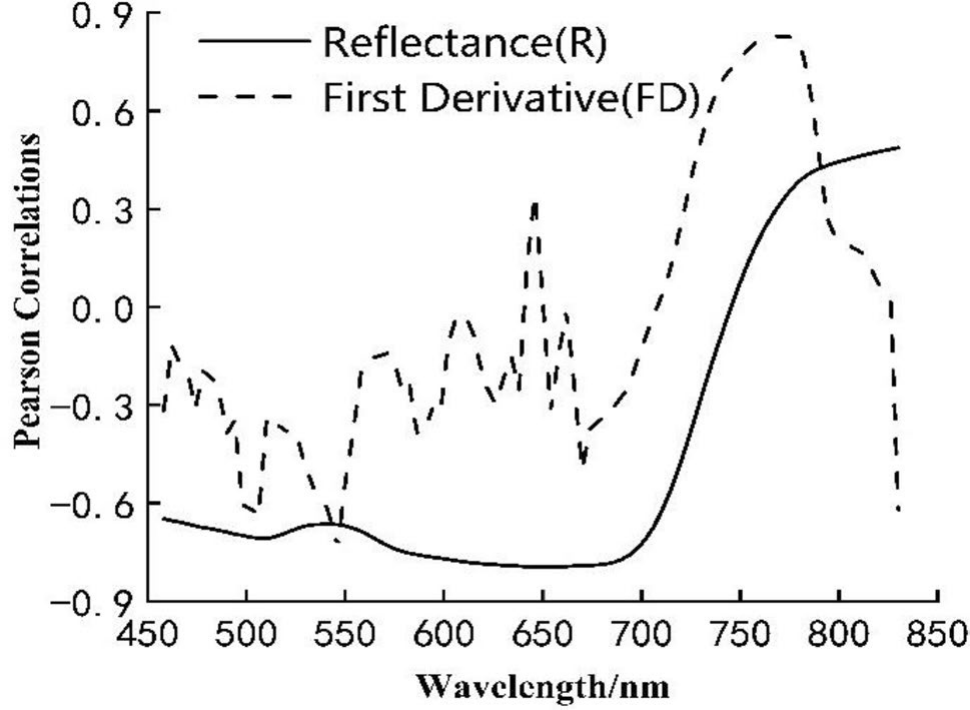
Correlation between UHD185 hyperspectral data and wheat LAI

Comparisons were made between the winter wheat LAI and raw UHD185 spectral reflectance (458–830 nm), and between LAI and FD-transformed UHD185 spectral reflectance (458–830 nm). Between LAI and UHD185 spectral reflectance, the maximum negative and positive correlation coefficients were at 654 nm (r = − 0.80) and 802 nm (r = 0.49), respectively (Fig. 6). Between LAI and FD-transformed spectral reflectance, the maximum negative and positive correlation coefficient were at 546 nm (r = − 0.74) and 774 nm (r = 0.83), respectively. Spectral bands with absolute correlation greater than 0.6 are 498–506 nm, 542 nm, 546 nm, 738–786 nm, and 830 nm (Fig. 6).

### Selection of characteristic bands related to winter wheat LAI from UAV hyperspectral reflectance data

Four methods including FD, SPA, CARS, and CARS_SPA were used to select characteristic bands related to wheat LAI. The FD correlation analysis demonstrated that spectral bands of 498–506 nm, 542 nm, 546 nm, 738–786 nm, and 830 nm were in high correlation with winter wheat LAI (correlation coefficients > 0.6). We selected bands corresponding to the maximum value at the inflection point, and discarded multiple highly correlated bands that are close to the inflection point. In this way, spectral bands at 506, 546, 774, and 830 nm were selected by FD, which account for 4.25% of the total variables.

**Fig. 7:**
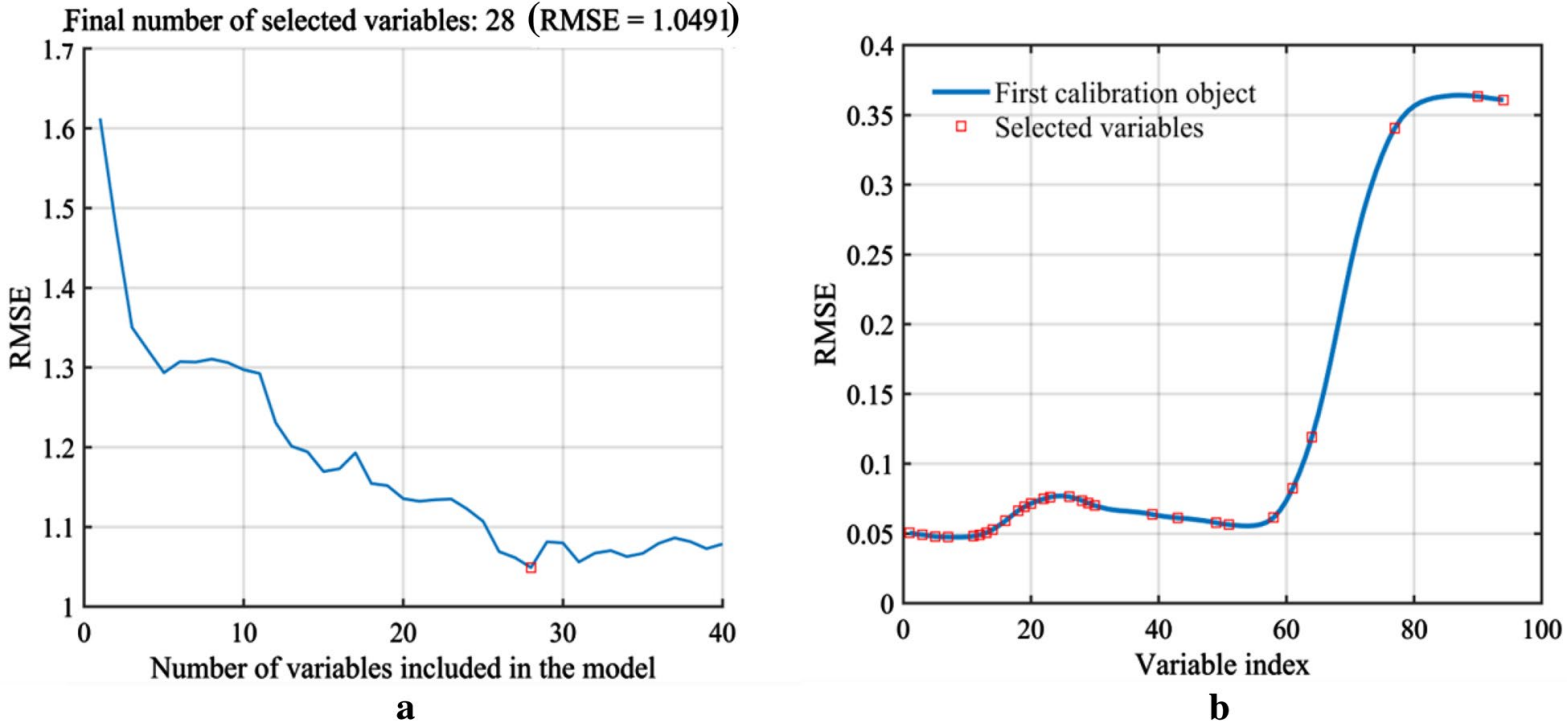
SPA Variable filtering process. **a** Change of the root mean square error (RMSE) in the SPA. **b** The optimal wavebands selected using SPA

During the process of SPA algorithm, the minimal and maximal numbers of characteristic bands extracted were set as 5 and 94, respectively. At the minimum RMSE of 1.049, a total of 28 optimal characteristic bands (458, 466, 474, 482, 498, 502, 506, 510, 518, 526, 530, 534, 542, 546, 558, 566, 570, 574, 610, 626, 650, 658, 686, 698, 710, 762, 814, and 830 nm), accounting for 29.8% of the total variables, were selected (Fig. 7).

**Fig. 8:**
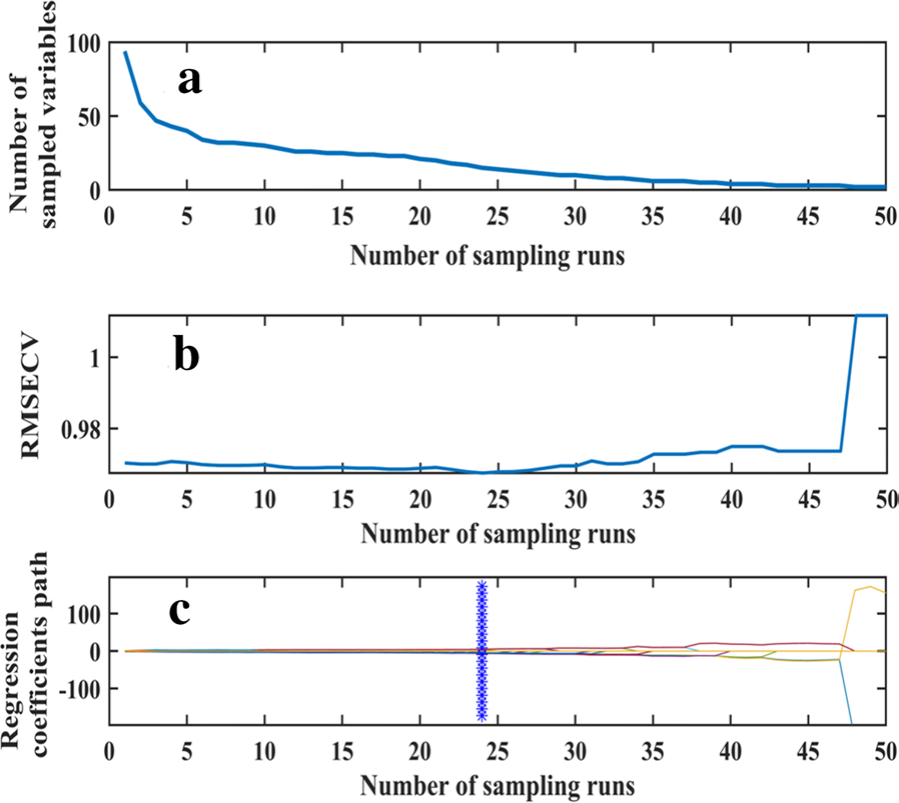
Selection of variables by CARS. **a** Variation trend of variables; **b** Tenfold RMSEV values; **c** Variable regression coefficient

The number of characteristic bands gradually decreased with the increase in CARS iteration (Fig. 8a). With the increase in sampling time, the tenfold RMSE cross validation first slightly decreased, then increased significantly at the CARS iteration of 47 (Fig. 8b). This result indicates that certain key information was lost after performing 47 iterations of CARS, resulting to a poor performance of the model. At the iteration of 24, RMSE reached its minimum of 0.9674. Thirteen variables (566, 586, 602, 610, 634, 682, 698, 710, 730, 734, 790, 802, and 814 nm) accounting for 13.8% of the total variables were therefore selected (Fig. 8c).

**Fig. 9:**
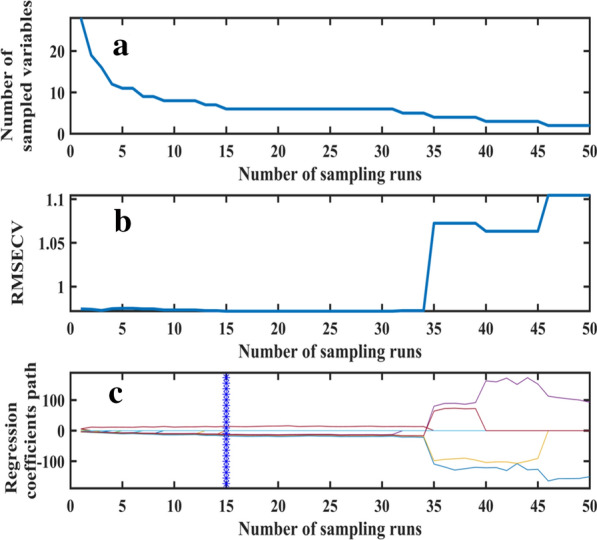
Variable selection process by CARS_SPA. **a** Variation trend of variables; **b** Tenfold RMSEV values; **c** Variable regression coefficient

The 28 characteristic bands selected using SPA algorithm may contain noise due to the complexity in SPA calculation process CARS algorithm can effectively remove the variables with small weight, and effectively select variables closely related to LAI. Therefore, the 28 characteristic bands selected by SPA algorithm were filtered by CARS algorithm to obtain the optimal combination of characteristic bands. The result showed the minimum RMSE (0.9718) appeared at the CARS iterations of 15 (Fig. 9). Therefore, nine variables (466, 474, 518, 526, 610, 658, 710, 814, and 830 nm) accounting for 9.57% of the total variables were selected.

**Fig. 10:**
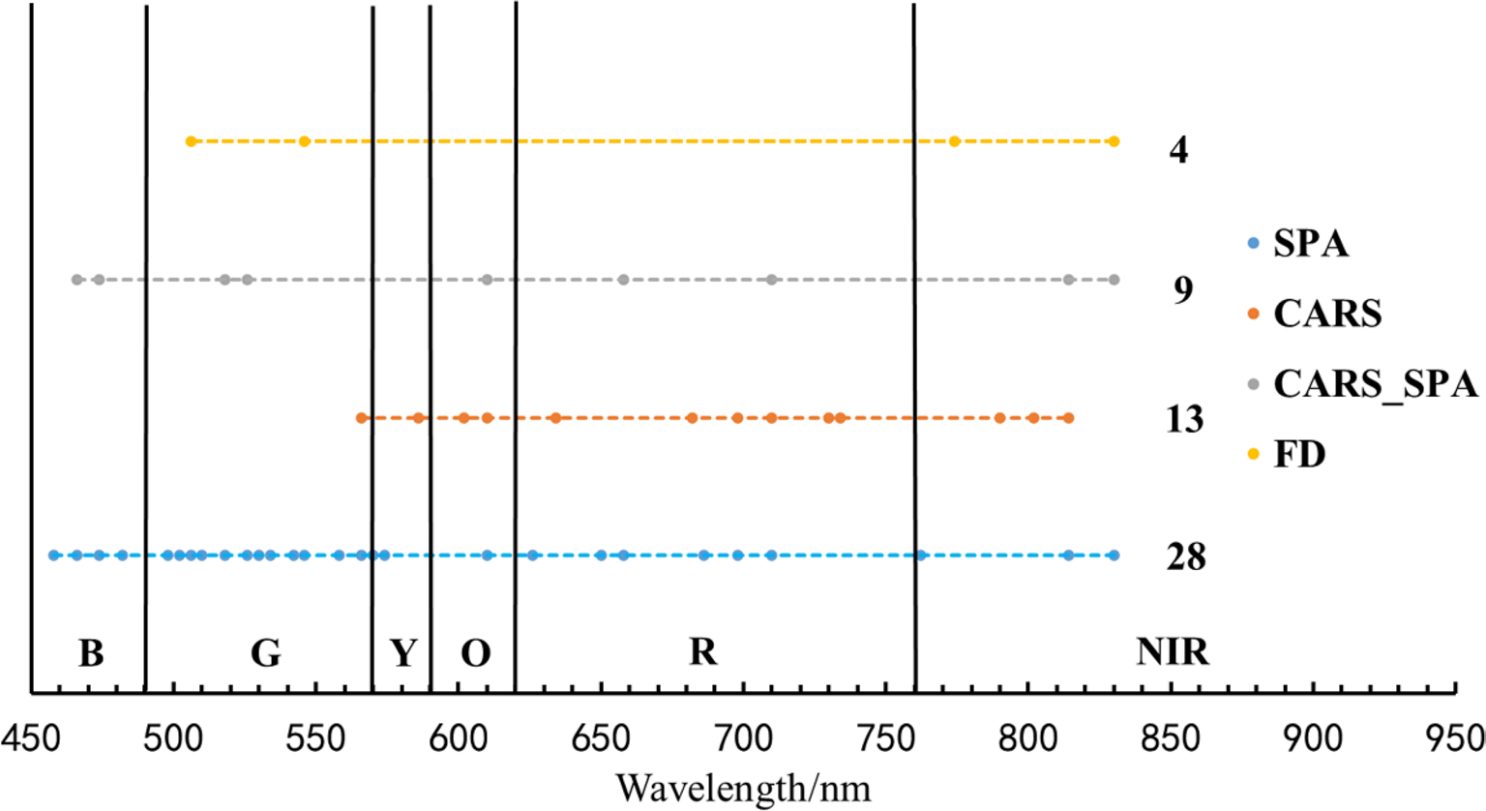
Distribution of characteristic bands selected by using different variable extraction algorithms. *SPA* Successive projections algorithm, *CARS* Competitive adaptive reweighed sampling, *CARS_SPA* competitive adaptive reweighed sampling combined with successive projections algorithm, *FD* First derivative, Reflectance: The original reflectivity curve. *B* Blue, *G* Green, *Y* Yellow, *O* Orange, *R* Red, *NIR* Near-infrared

We compared the LAI characteristic bands selected by the four algorithms, the distributions of characteristic bands selected by different algorithms were in consistent to a large extent, yet differences were also observed (Fig. 10).

### Construction of winter wheat LAI estimation model by different modeling methods

Based on characteristic bands of wheat LAI selected by using different variable selection algorithms and full spectrum information, three modeling methods including PLSR, SVR, and Xgboost were employed to construct LAI estimation models. Independent samples including calibration and validation sets were used to test these models. Table 2 summarizes the results for the models obtained by different modeling methods.

**Table 2:** Regression analysis of characteristic bands and winter wheat LAI

Modeling method	Variable extraction	Wavelengths numbers	Calibration			Validation		
			R^2^	RMSE	RPD	R^2^	RMSE	RPD
PLSR	Full_spectrum	94	0.79	0.81	2.15	0.78	0.79	2.06
	FD	4	0.81	0.77	2.26	0.81	0.75	2.15
	SPA	28	0.79	0.81	2.14	0.78	0.81	2.01
	CARS	13	0.80	0.79	2.22	0.81	0.78	2.08
	CARS_SPA	9	0.83	0.73	2.39	0.83	0.74	2.19
SVR	Full_spectrum	94	0.80	0.79	2.20	0.77	0.79	2.06
	FD	4	0.80	0.78	2.22	0.80	0.72	2.24
	SPA	28	0.81	0.75	2.31	0.79	0.76	2.14
	CARS	13	0.79	0.79	2.20	0.77	0.79	2.04
	CARS_SPA	9	0.82	0.75	2.31	0.84	0.65	2.75
Xgboost	Full_spectrum	94	0.93	0.50	3.48	0.80	0.79	2.04
	FD	4	0.88	0.65	2.69	0.81	0.75	2.17
	SPA	28	0.84	0.73	2.37	0.76	0.82	1.96
	CARS	13	0.82	0.82	2.12	0.82	0.84	1.93
	CARS_SPA	9	0.89	0.63	2.51	0.89	0.55	2.92

A reliable PLSR model was constructed using 9 characteristic bands selected by CARS_SPA algorithm as input. In this model, similar results of model evaluation indices (*R*^*2*^, RMSE, and RPD) were obtained for the calibration and validation sets. The *R*^*2*^, RMSE, and RPD of the calibration set were 0.83, 0.73, and 2.39, respectively, and those of the validation set were 0.83, 0.74, and 2.19, respectively. The PLSR model constructed based on 28 characteristic bands selected by SPA algorithm showed a poor performance. The *R*^*2*^, RMSE, and RPD of the calibration set were 0.79, 0.81, and 2.14, respectively, and those of the validation set were 0.78, 0.81, and 12.01, respectively.

The SVR method based on different combinations of characteristic bands yield similar results of evaluation indices. Among these SVR models, the one using 9 characteristic bands selected by CARS_SPA algorithm as input showed the best performance. The *R*^*2*^, RMSE, and RPD of the calibration set were 0.82, 0.75, and 2.31, and those of the validation set were 0.84, 0.65, and 2.75.

Further analysis was performed on the model constructed by using Xgboost method. The Xgboost model based on the 28 characteristic bands selected by SPA algorithm showed a poor performance. The *R*^*2*^, RMSE, and RPD of the calibration set were 0.84, 0.73, and 2.37, respectively, and those of the validation set were 0.76, 0.82, and 1.96, respectively. The Xgboost model showed the best performance when using 9 characteristic bands selected by CARS_SPA algorithm. The *R*^*2*^, RMSE, and RPD of the calibration set were 0.89, 0.63, and 2.51, respectively, and those of the validation set were 0.89, 0.55, and 2.92, respectively.

**Fig. 11:**
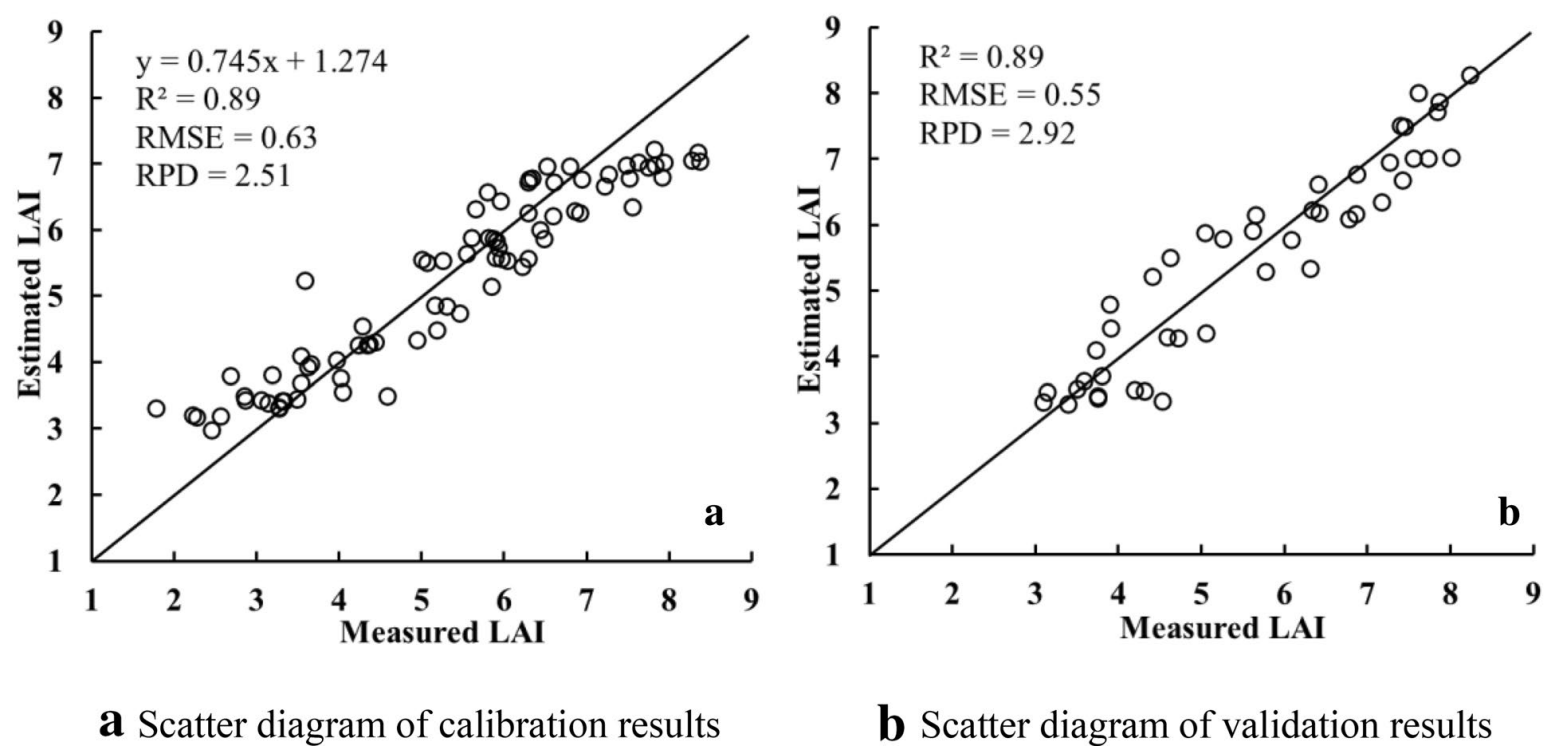
Calibration and validation results of best-performed wheat LAI estimation model. **a** Scatter diagram of calibration results. **b** Scatter diagram of validation results

**Fig. 12:**
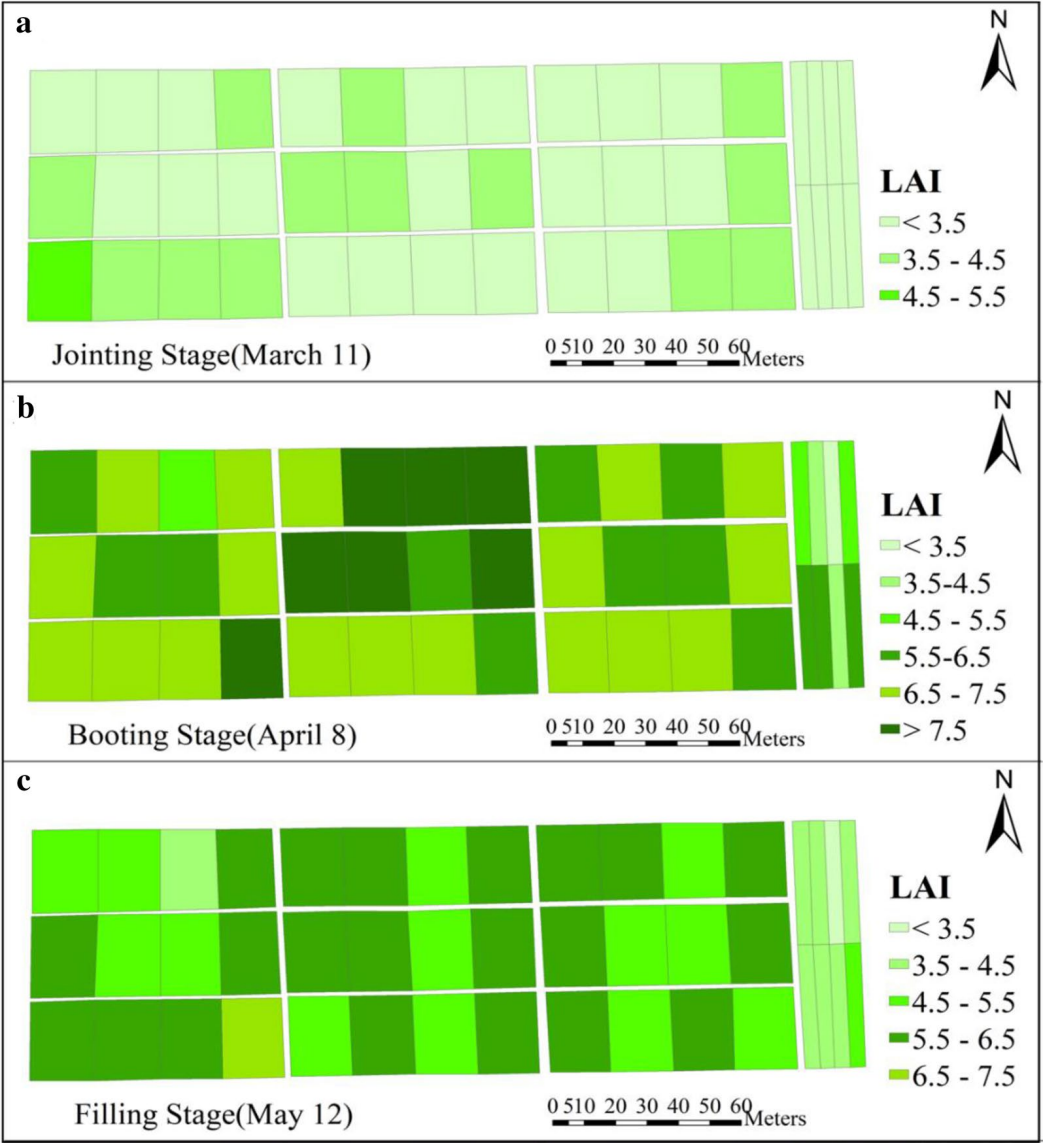
Spatial distribution diagram of LAI estimation based on wheat LAI estimation model

In summary, among all characteristic band combinations, the one containing 9 characteristic bands selected by CARS_SPA algorithm outperformed with either of the ML modeling methods, followed by models constructed using the four characteristic bands selected by FD algorithm. This may be due to the fact that these 9 characteristic bands selected by CARS_SPA algorithm are uniformly distributed within the spectral range of 458–830 nm, which thus well maintain the spectral information of the reflectance corresponding to LAI inversion. The three LAI estimation models constructed based on characteristic bands selected by CARS_SPA were superior to LAI models constructed based on the full spectrum. However, the accuracies of LAI models constructed based on selected characteristic band were different from those of the models constructed based on full spectrum information. These results demonstrated that characteristic bands extraction could greatly reduce the number of variables used for modeling, thus reduces the modeling complexity which improves the modeling efficiency while ensures its accuracy. Comparing the three ML modeling methods, the Xgboost models performed the best, followed by PLSR and SVR. The calibration and validation results of the best-performed model are shown in Fig. 11. Figure 12 shows the predicted LAI at the jointing, booting, and filling stages based on the 9 characteristic bands selected via the CARS_SPA algorithm and the winter wheat LAI model constructed by XGBoost. The mapping was performed by combining UAV images, with which the spatial variation of LAI can be visualized.

## Discussion

In combine with VI or other modeling methods, a large number of wheat LAI estimation models have been built based on selected characteristic bands related to wheat LAI. However, the characteristic bands used in different studies were quite varied. For example, Cheng et al. [32] quantitatively analyzed UAV hyperspectral data, and found that 516, 636, 702, 760, and 867 nm were the most sensitive bands to LAI changes. Gao et al. [33] constructed a ratio VI model for estimating wheat LAI using UAV hyperspectral data of 494 and 610 nm, resulting to a modeling *R*^*2*^ of 0.74 and a prediction *R*^*2*^ of 0.78. Xie et al. [12] used airborne hyperspectral data to study the normalized VI and found that models constructed at 660 and 785 nm show better estimation winter wheat LAI. By using the hyperspectral data of UAV, Tao et al. [34] confirmed that the linear combination index (LCI) constructed at 670, 710, and 850 nm and the plant biochemical index (PBI) constructed at 560 and 810 nm were coupled with red edge parameters respectively, which can be used to accurately estimate winter wheat LAI. Based on the UAV hyperspectral data and optimal index algorithm, Chen et al. [35] determined 454, 754, and 834 nm were the optimal bands for winter wheat LAI estimation model during the flowering period. The red and near-infrared bands used in the above studies can better reflect the dynamic changes of LAI [36]. Bands of 518, 610, 658, 710, 810, and 830 nm selected in this study are similar to those in the aforementioned studies. In addition, the 9 characteristic bands selected by CARS_SPA algorithm in this study also include 466, 474, and 526 nm, which are the convoluted absorption bands of chlorophyll and carotenoids [37].These bands are in the blue-green light spectral range, which would perform better in LAI prediction if combined with red and near-infrared bands [38, 39]. The three models built based on the 9 characteristic bands selected by CARS_SPA algorithm all showed outstanding prediction results, indicating that the nine bands may contain effective information related to winter wheat LAI. These results may provide reference for other related studies.

The ML methodologies can be used to effectively analyze and utilize information-rich datasets as well as high-dimension observation data [40]. It has been used in the analysis and modeling of remote sensing data, but the accuracies of different ML methods are varied. Based on UAV hyperspectral data, Tao et al. [34] combined the VI and red edge parameters, and constructed an estimation model of winter wheat LAI using PLSR method. This model yielded a modeling *R*^*2*^ of 0.80, and a prediction *R*^*2*^ of 0.75. Based on UAV hyperspectral data, Tao et al. [41] built a winter wheat LAI inversion model in combination with multiple linear regression of random forest in the flowering period, which performed the best, resulting to a modeling *R*^*2*^ of 0.68, and a prediction *R*^*2*^ of 0.85. In this study, three ML methods were employed to construct winter wheat LAI estimation model. In general, the Xgboost models perform the best. This may be due to the fact that Xgboost is fast, highly effective, suitable for large-scale data processing, and has custom loss function [30], Xgboost can tackle majority of the flaws that emerged from the existing modeling methods [42]. In this study, the Xgboost model built based on the 9 characteristic bands selected by the CARS_SPA algorithm has been proved to be the best model for estimating winter wheat LAI, the accuracy of which (calibration *R*^*2*^ = 0.89; validation *R*^*2*^ = 0.89) is higher than those constructed by Tao et al. [34, 41]. Therefore, Xgboost may be a reliable modeling method, which can be used for UAV remote sensing in combine with wheat LAI modeling and prediction. The findings in this study provide technical support for rapid and nondestructive estimation of winter wheat LAI. However, this study only compared the potential of three machine learning methods for UAV estimation of wheat LAI, while deep learning showed better application prospects in areas such as classification and monitoring of plant pest and disease [43, 44], and it is necessary to further explore the potential of deep learning and other methods for UAV spectral monitoring of crop growth in the next step. In addition, more samples need to be obtained combine with multi-point and multi-year experiments, and a more universal and applicable model will be established on this basis.

## Conclusion

Based on different nitrogen treatments and field experiments, hyperspectral images, and LAI at key growth stages of winter wheat were obtained by the UAV platform equipped with hyperspectral imaging sensor. Four algorithms were used to extract characteristic bands related to LAI, and three ML methods were used to construct models for estimating winter wheat LAI. We compared the reliability and accuracy of these models, and found out that the Xgboost model constructed based on 9 characteristic bands selected by CARS_SPA algorithm exhibited the highest accuracy. In this model, the *R*^*2*^, RMSE, and RPD of the calibration set were 0.89, 0.63, and 2.51, respectively, and those of the validation set were 0.89, 0.55, and 2.92, respectively. The combination of CARS_SPA algorithm and Xgboost modeling method can reduce input variables, improve the operation efficiency of the model, and ensure a higher accuracy. Our results provide a reference for the nondestructive and rapid acquisition of winter wheat LAI by UAV remote sensing.

## Data Availability

The data sets used and/or analyzed during the current study available from the corresponding author on reasonable request.
